# Omilayers: a Python package for efficient data management to support multi-omic analysis

**DOI:** 10.1186/s12859-025-06067-7

**Published:** 2025-02-06

**Authors:** Dimitrios Kioroglou

**Affiliations:** Integrative Genomics Lab, Center for Cooperative Research in Biosciences (CIC bioGUNE), Basque Research and Technology Alliance (BRTA), Bizkaia Technology Park, Derio, Basque Country Spain

**Keywords:** Multi-omics, Data management, Databases, Python

## Abstract

Multi-omic integration involves the management of diverse omic datasets. Conducting an effective analysis of these datasets necessitates a data management system that meets a specific set of requirements, such as rapid storage and retrieval of data with varying numbers of features and mixed data-types, ensurance of reliable and secure database transactions, extension of stored data row and column-wise and facilitation of data distribution. SQLite and DuckDB are embedded databases that fulfil these requirements. However, they utilize the structured query language (SQL) that hinders their implementation by the uninitiated user, and complicates their use in repetitive tasks due to the necessity of writing SQL queries. This study offers Omilayers, a Python package that encapsulates these two databases and exposes a subset of their functionality that is geared towards frequent and repetitive analytical procedures. Synthetic data were used to demonstrate the use of Omilayers and compare the performance of SQLite and DuckDB.

**Supplementary Information:**

The online version contains supplementary material available at 10.1186/s12859-025-06067-7.

## Introduction

Multi-omic analysis encompasses the thorough evaluation and integration of numerous and diverse biological features utilizing high-throughput technologies that generate omic layers known as genomics, metabolomics, transcriptomics and proteomics, among others. Each omic layer offers unique insights to unravel the underlying complexity that governs a given phenotype, empowering medicine to move towards a more personalized direction and provide greater precision regarding the identification of phenotypic biomarkers and choice of treatment. Thus, the ultimate goal of multi-omic analysis is the identification of latent patterns to allow patients stratification and lead to targeted treatments. However, the realization of this goal involves many challenges including data management and privacy, gaps of knowledge on biological functions and patient participation and sample size of available omic data [1]. The current study focuses on data management and aims to provide a solution to simplify the storage, retrieval, and distribution of multi-omic datasets.

Currently there are numerous databases each one providing its own unique interface and query language to store and retrieve data. Since it is difficult to satisfy all use cases, each database solution is optimized for a specific architecture requiring the user to learn a new syntax and proceed to code adaptation when switching between databases. Python packages, such as SQLAlchemy [2], try to solve this issue by providing object-relational mappers (ORM) that add a layer of abstraction that allows the user to utilize the same Python code and interact with different databases. However, the user needs to manually define Python classes and map them to corresponding database tables, rendering difficult the implementation of ORM solutions to diverse datasets.

**Fig. 1:**
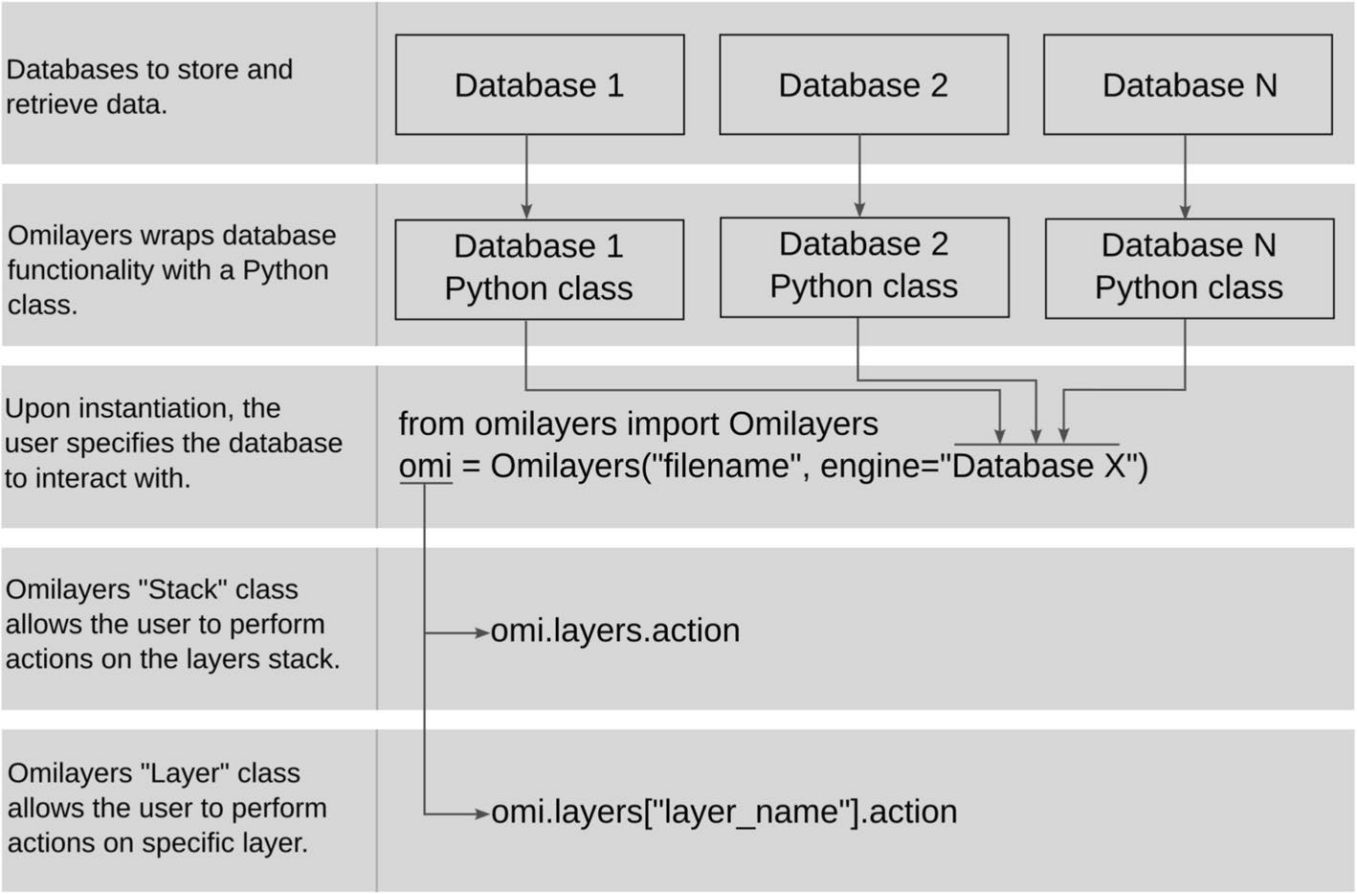
Diagram illustrating the core rationale behind Omilayers' design and functionality. Omilayers is built to encapsulate the functionality of one or more databases within a dedicated Python class. This design allows users to seamlessly interact with different databases while using the same consistent set of actions for storing, retrieving, updating and extending data, regardless of the chosen database. Omilayers provides functionality at two levels: Stack-level operations and Layer-level operations. The former exposes actions across all stored layers, accessible via the predefined Stack class, and the latter exposes actions specific to a single layer, available through the predefined Layer class

Omilayers follows a similar rationale but eliminates the need for a user to define Python classes and provides a simplified application programming interface (API) to interact with a given database. The functionality of a given database is encapsulated by Omilayers within dedicated Python classes, allowing the user to simply choose which database to interact with when creating an instance of an Omilayers object (Fig. 1). Then, Omilayers exposes a given set of methods through the predefined Stack and Layer classes, allowing the user to store, retrieve and alter data using the same Python code regardless the chosen database.

Since the task to encapsulate a given database is being undertaken by Omilayers, it was necessary to establish a set of criteria for selecting which databases to include. First, to effectively manage multi-omic datasets it is crucial to be able to store data with varying numbers of features. Moreover, rapid data retrieval is necessary to meet the demands of timely data analysis. Currently, there are matrix-based data manipulation Python packages that meet these two requirements, such as AnnData [3] and Xarray [4]. However, they insufficiently address requirements such as extension of stored data row and column-wise, storage of data with mixed data-types and full ACID (atomicity, consistency, isolation, and durability) compliance. The latter is crucial in order to ensure that the data remain consistent and accurate even during system failures.

Relational database management systems (RDBMS) can satisfy all the above criteria. They store data in data-structures called tables, and use the structured query language (SQL) to access and manipulate the stored data. PostgreSQL and MySQL are popular and well-tested RDBMS, however they are server-based RDBMS and as consequence they violate the requirements of an in-process database and the storage of data in a single file. These two requirements are crucial to avoid elaborate database configurations, and allow portability and efficient distribution of the stored data.

After considering all the above criteria, Omilayers encapsulates the functionality of SQLite [5] and DuckDB [6]. Both databases represent great solutions for analytics with SQLite having the advantage of being part of the Python standard library through the sqlite3 module, and DuckDB being designed for high-performance data transfer. Since both are SQL databases, Omilayers wraps in a dedicated Python class a subset of their capabilities that is suitable for frequent and repetitive interactions. Thus, the user can store, retrieve and alter data without the need to write SQL queries. Moreover, Omilayers API resembles the Pandas API [7] to facilitate usage and enhance code readability.

In the current study synthetic omic layers are used to exhibit the usability of Omilayers for data analysis and compare the performance of the included databases during data storage and retrieval.

## Methods

### Machine specifications

All interactions with each database have been performed in a single-threaded mode on a typical laptop with a i7-10510U processor (4 cores, 2 threads each) clocked at 1.80 GHz, 32 GB memory, 1 TB storage and running 64-bit Rocky Linux 8.9.

### Simulation of synthetic cohort characteristics

The simulated cohort encompasses 100 samples and a layer was created to include cohort characteristics such as sample ID, gender, age and BMI. A uniform distribution was used to generate integer values for age (range from 20 to 50) and 2-decimal float values for BMI (range from 20 to 40). This layers was stored under the name "cohort" in the databases.

### Simulation of synthetic metabolomic data

The synthetic metabolomic data include metabolites from blood and urine. Corresponding metabolites were downloaded from the human metabolome database (HMDB) [8] and a uniform distribution, with range from 0 to 1000, was used to generate an array of 100 4-decimal float values for each metabolite. The synthetic blood and urine layers were stored under the names “blood_metas” and “urine_metas” respectively in the databases.

### Simulation of synthetic bulk RNASeq transcriptomic data

The GENCODE portal [9] was used to extract gene ENSEMBL IDs from the GFF3 annotation file of the human genome (GRCh38.p14). Then a uniform distribution, with a range from 0 to 1000, was used to generate an array of 100 integer values for each gene. This layer was stored under the name “rnaseq” in the databases.

### Simulation of synthetic gut microbiome data

The GMrepo portal [10] was used to download human gut microbiome data at species level. A uniform distribution was then used, with range from 0 to 1000, to generate an array of 100 integer values for each species. This layer was stored under the name "microbiome" in the databases.

### Simulation of synthetic germline short variants from DNA sequencing data

An in-house variant call format (VCF) file was available from a human cohort that provided whole-exome and genotypes from genotypic arrays (GSA) for an independent study. The whole-exome data were previously processed following the GATK best practises pipeline for germline short variants identification and merged afterwards with the GSA data. The merged genomic data were then imputed using the Michigan Imputation Server (MIS) [11].

In order to simulate synthetic genomic data, the total number of variants were counted for each chromosome from the in-house VCF. Then a synthetic VCF file was created where, starting from position 100,000, genomic positions in each chromosome were unit-incremented recursively till the corresponding total number of variants was reached. Uniform distributions were then used to generate values for alleles, sample genotypes and metrics returned from the GATK and MIS pipelines. This layer was stored under the name “vcf” in the databases.

This layer was the only layer whose synthetic data were not retained on memory but written in a file. The latter occupied a disk space of 37 GB when uncompressed and 12 GB when compressed.

### Code used for simulation and benchmarks

Detailed Python code on the simulations and timing of the interactions with the database is given in the Github repository of Omilayers that includes a jupyter notebook for SQLite (https://github.com/dkioroglou/omilayers/blob/main/synthetic_data/synthetic_omic_data_sqlite.ipynb) and one for DuckDB (https://github.com/dkioroglou/omilayers/blob/main/synthetic_data/synthetic_omic_data_duckdb.ipynb).

### Documentation on Omilayers usage

In the current manuscript an implementation section is provided that describes the basic usage of Omilayers. The full documentation on Omilayers usage is hosted on https://omilayers.readthedocs.io/en/latest/ where it will be updated in each release.

## Implementation

Initially, the user needs to create an Omilayers object and specify the database to interact with (Code snippet 1). If no database is specified, DuckDB is used by default. Then, all commands to store, retrieve and alter the stored data are the same regardless the chosen database.
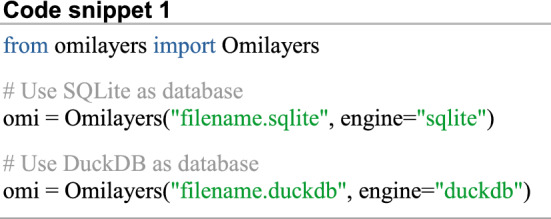


Omilayers depends on Pandas and thus each synthetic omic layer was created as a Pandas dataframe object (supplementary methods S1) and stored to the database as shown in the Code snippet 2.
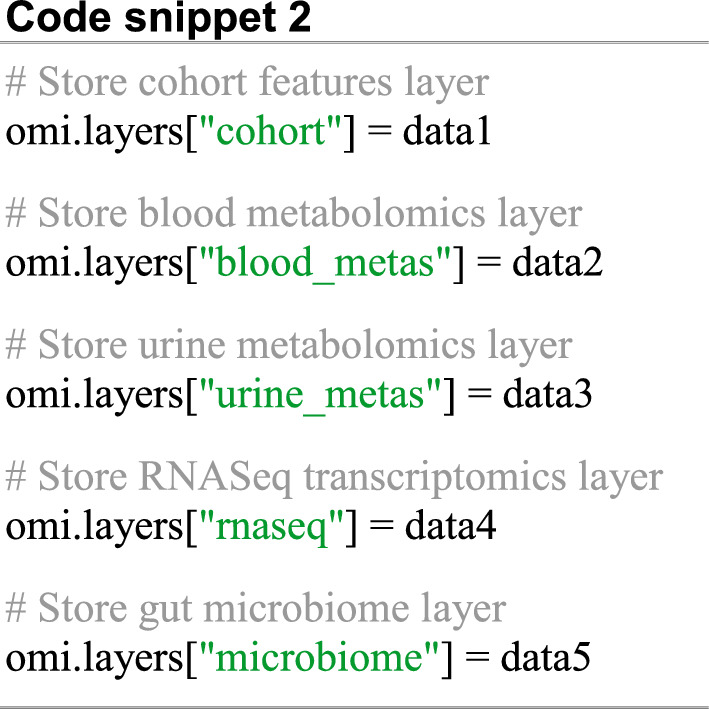


To store the synthetic VCF layer, the file was not loaded entirely on memory as the other omic layers, but instead it was loaded and stored in chunks of 100,000 lines from its compressed form (Code snippet 3).
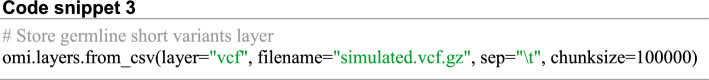


Upon storage, the user can annotate the stored layer by providing an arbitrary tag name and description (Code snippet 4).
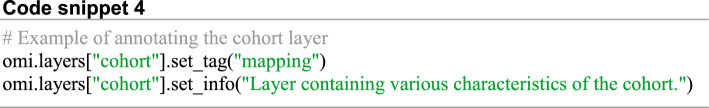


The tag name is useful for grouping layers together. This way the user can retrieve the names of all stored layers that have been annotated with the same tag name (Code snippet 5).
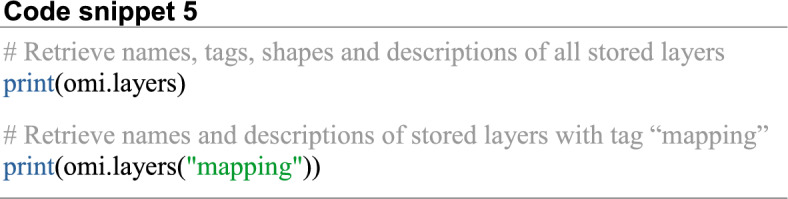


Regarding data retrieval, the user can either retrieve information on the stored data of a given layer, load the entire stored layer on memory, load all rows for a given column or load data based on row values (Code snippet 6).
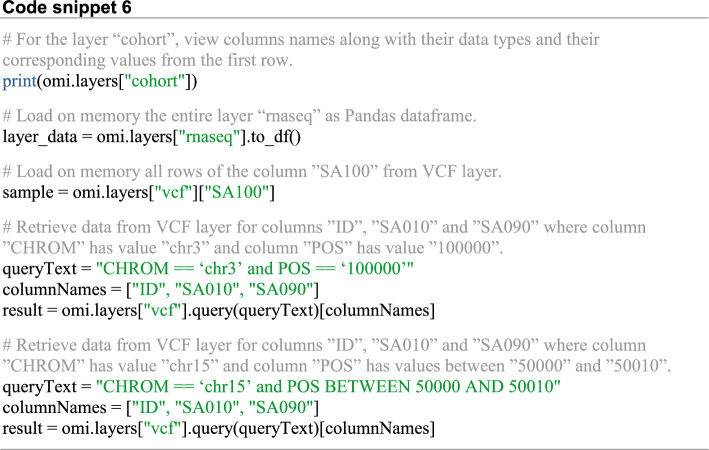


To alter the stored layers, the user can extend a given stored layer column or row-wise using new data in the form of a Pandas dataframe (Code snippet 7).
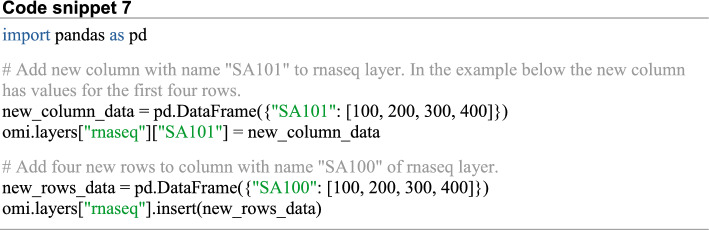


To join one or more stored omic layers, they should have one column in common. If this condition is met, then data from the omic layers can be retrieved with Omilayers as Pandas dataframes and joined by Pandas (Code snippet 8).
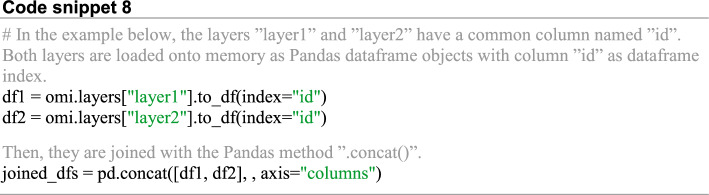


## Results

### Short runtimes were observed during storage and data retrieval for both databases

**Fig. 2:**
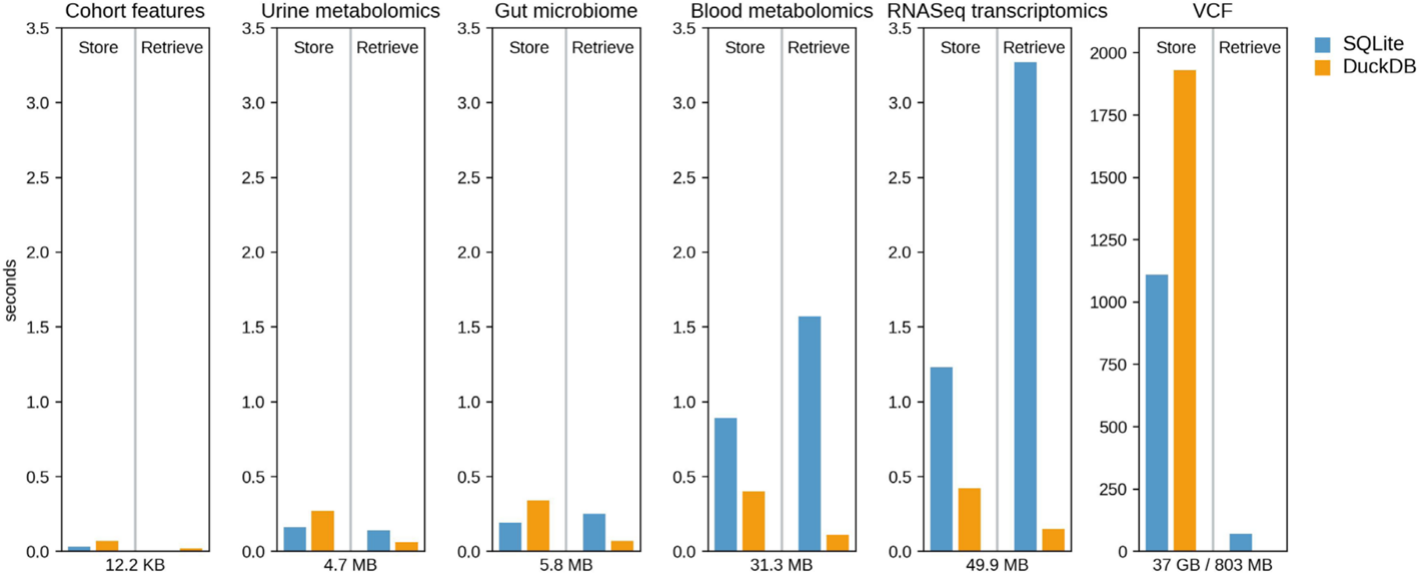
Results showing the runtime in seconds of SQLite and DuckDB during data storage and retrieval, along with the name and memory footpring of the corresponding synthetic dataset. For all datasets the same data size was stored and retrieved, except the VCF layer. For the latter, the file of size 37 GB was loaded in chucks of 100,000 during storage, and the data retrieval concerned only a single column with a memory footpring of 803 MB

Figure 2 shows the runtime in seconds to store the synthetic omic layers using either SQLite or DuckDB. Both databases exhibited very good performance during data storage with runtimes suitable for analytical purposes, even for the synthetic VCF layer that required 30 h to be generated. More specifically, SQLite was slightly faster than DuckDB for small datasets but performed slower as the dataset became bigger. Interestingly, this relationship was not observed for the VCF layer, where DuckDB was almost two-fold slower than SQLite. Since this was the only layer that was stored in chunks, the observed performance indicates that DuckDB is less optimized than SQLite for storing data in small and frequent transactions that need to read and write to disk. To summarize the observed performance of the two databases, a linear regression without intercept was performed, using time to store the omic layer as response variable and data size as independent variable. The results showed that as the data size increases by 1 megabyte (MB) the time to store the data increases by 25 ms for SQLite (95% CI 23–28 ms) and 10 ms for DuckDB (95% CI 1–19 ms). Note that in the linear regression the time for storing the VCF layer was not considered since this step included the additional factor of multiple disk reads and writes.

For comparing data retrieval runtimes, the entire omic layers were loaded on memory except the VCF layer where all stored variants for a given sample were parsed (supplementary methods S2). The latter resulted in a memory footprint of 803 MB. Overall, DuckDB displayed better performance than SQLite across all data sizes (Fig. 2). After performing a linear regression in the same way as described above and considering also the results from the VCF layer, it was observed that the time to retrieve the stored data increases by 88 ms for SQLite (95% CI 83–92 ms) and 2.3 ms for DuckDB (95% CI 2.1–2.4 ms) for every 1 MB increase of the data size. This difference in data retrieval speed can be attributed to the fact that DuckDB is column-based database, whereas SQLite is a row-based database. This allows DuckDB to perform vectorized executions that permit data processing in large batches than row-by-row.

### DuckDB displayed higher efficiency regarding disk space usage than SQLite

Another benefit of column-based databases is that a lightweight compression on the stored data can be applied. Without considering the VCF layer, the generation of the rest of the layers resulted in a total memory footprint of 93 MB that translated into 67 MB of total disk usage after storage for SQLite and 66 MB for DuckDB. However, the difference between the two databases increased drastically after storing the VCF layer. More specifically, the size of the SQLite increased to 37 GB upon storage, whereas DuckDB with an increase of 13 GB remained close to the size of the compressed VCF file. This efficiency in data storage provides a great advantage to DuckDB since data analysis will generate more layers that need to be stored.

### DuckDB outperformed SQLite during addition of new column and row-based data retrieval

Extending the stored data column-wise involves changes to the file structure and represents a resource-intensive operation for most databases. Focus was given only to the transcriptomic and VCF layers due to their size, where data from columns were retrieved and added as new columns in order to compare the databases performance (supplementary methods S3). The added column included 60,649 rows for the transcriptomic layer (473.6 KB) and 9,640,953 rows (803 MB) for the VCF layer. SQLite displayed a runtime of 520 ms for the former and 316.49 s for the latter layer, compared to DuckDB’s runtime of 140 ms and 9.87 s respectively. Once again the two databases exhibited great difference in disk space with SQLite increasing by 27 GB its size and DuckDB only by 550 MB.

Following the above observations on column-based operations, the next step was to evaluate the performance of the databases on row-based queries. For this only the VCF layer was used where the data for a given set of columns were parsed for different positions (supplementary methods S4). On average SQLite returned results in 33.07 s (SD = 0.31 s), whereas DuckDB in 0.10 s (SD = 0.008 s). The difference in performance increased even further during the execution of 297 queries utilizing 8 cores in parallel, with SQLite marking a runtime of 1800s and DuckDB 8.47 s (supplementary methods S5).

## Discussion

Multi-omic integration involves the processing and analysis of diverse omic data and represents a holistic approach aiming at enhancing the understanding of complex biological processes associated with a given phenotype. This study offers the Python package Omilayers as a solution to facilate bioinformatic analysis and management of multi-omic data. At its core, Omilayers encapsulates the APIs of SQLite and DuckDB and exposes a subset of their functionality that is geared towards frequent analytical bioinformatic procedures. Although SQL is a straightforward language, it can become quite tedious to write and use for highly repetitive and frequent tasks as those involved in data analysis. Moreover, many SQL queries require adaptation to be used between SQLite and DuckDB. Thus, by providing a unified API that resembles the Pandas API, Omilayers eliminates the need to write SQL queries and learn the syntactic idiosyncrasies of each database.

Since Omilayers stores data from a Pandas dataframe object, the structure of the latter may either have omic features as columns and samples as rows or the opposite. However, SQLite supports only a few thousand columns per table, making it unsuitable for storing very large omic layers with features represented as columns. On the other hand, while DuckDB can theoretically handle nearly 2 billion columns per table, its column-based design is better suited for representing features as rows and samples as columns, optimizing sample retrieval performance. Thus, the recommended structure of the omic layer is to include samples as columns and features as rows.

Following the UNIX philosophy and to facilitate code maintenance, the functionality of the databases that has been exposed by Omilayers concerns only data management operations such as data storage, retrieval, update and extension (row and column-based). For other use cases that require operations on the database, the direct usage of the databases APIs is recommended.

Overall, DuckDB exhibited better performance than SQLite and is the default database Omilayers uses in case no database is specified by the user. The performance of SQLite during data retrieval could be optimized by creating an index for a given column. However, this requires additional disk space and can reduce performance during data extension, as the index must be updated accordingly. Nevertheless, for small to medium-sized datasets the user might choose SQLite over DuckDB for use cases that require frequent and small transactions with multiple reads and writes to disk. Moreover, SQLite is fully backwards compatible whereas DuckDB has added this feature since version v0.10. This means that versions of DuckDB prior to v0.10 cannot read data stored with different versions than the one they were created with.

Regarding limitations, DuckDB does not support distribution of workloads across multiple machines and writing from multiple processes without the use of a database lock. While this impedes high volume transactions, these constrains do not hinder multi-omic analysis that is conducted on a single machine. Moreover, the current study did not include the simulation of synthetic single-cell RNASeq transcriptomic data that represent sparse data. The development of bioinformatic analytical procedures for this omic layer has currently been undertaken by the Python framework Scanpy [12] that depends on AnnData for data management. Thus, storing these omic data with Omilayers would lead to unnecessary redundancy.

Regarding scalability, it is important to note that both included databases scale vertically, meaning performance bottlenecks caused by hardware limitations can only be mitigated by hardware upgrade. Moreover, since Omilayers can handle data that do not fit in memory, as shown with the VCF layer, the performance is linked to the runtime. This implies that the performance of Omilayers is being expressed as the runtime the user must wait until a given database operation becomes impractical for bioinformatic analysis. However, the threshold that renders a given database operation impractical varies depending on the specific use case and user requirements.

The dependency of Omilayers on Pandas has been incorporated due to the wide adoption of the latter for data analysis and to ensure robustness during data storage. Although the current study has utilized only synthetic data, Omilayers is capable of storing any data that can be read and loaded as Pandas dataframes. Furthermore, the Python’s standard unit testing framework was utilized to allow users to evaluate the reliability of Omilayers on their machines after installation (supplementary methods S6).

Finally, although Omilayers was developed having multi-omic data in mind, it can be used with any kind of data. As a future work, more databases can be included in Omilayers, as long as they meet the established requirements and outperform the included databases.

## Supplementary Information

Supplementary Material 1.

## Data Availability

The compressed synthetic VCF file is available at https://doi.org/10.5281/zenodo.12790872. The omic features used to simulate the omic layers are available on the Github repository of Omilayers https://github.com/dkioroglou/omilayers/tree/main/synthetic_data/omic_features.

## Abbreviations

ACID: Atomicity, consistency, isolation, and durability
RDBMS: Relational database management systems
SQL: Structured query language
API: Application programming interface
