# Supplementary material for: Omilayers: a Python package for efficient data management to support multi-omic analysis
Source: BMC Bioinformatics. 2025 Feb 6;26:40. doi: 10.1186/s12859-025-06067-7 (PMC11800426; doi:10.1186/s12859-025-06067-7)
Supplement: Supplementary file 1 — Supplementary Material 1. [file 12859_2025_6067_MOESM1_ESM.pdf]

Supplementary methods

S1 Creation of synthetic data

All CSV and TXT files that are used in the code snippets below can be found in Omilayers Github repository: `https://github.com/dkioroglou/omilayers/tree/main/synthetic_data/omic_features`

Creation of data for the layer of Cohort features:

```python
import pandas as pd
import numpy as np

Nsamples = 100
sampleIDs = ["SA"+f"{i}".zfill(3) for i in range(1, Nsamples+1)]
data1= pd.DataFrame({
    "sample_id": sampleIDs,
    "gender": np.random.choice(["female", "male"], Nsamples),
    "age": np.random.randint(20, 50, Nsamples),
    "bmi": np.round(np.random.uniform(20, 40, Nsamples), 2)
})
```

Helper function for creating synthetic omic data:

```python
def simulate_data(featuresName, features, minValue, maxValue, integers=False):
    JSON = {}
    for feature in features:
        if not integers:
            JSON[feature] = np.round(np.random.uniform(minValue, maxValue, Nsamples), 4)
        else:
            JSON[feature] = np.random.randint(minValue, maxValue, Nsamples)
    data = pd.DataFrame(JSON, index=sampleIDs)
    data = data.T
    data = data.reset_index()
    data = data.rename(columns={"index":featuresName})
    return data
```

Creation of synthetic blood metabolomic data:

```python
features = pd.read_csv("omic_features/blood_metabolites-2024-06-25.csv")['NAME']
data2 = simulate_data("metabolite", features, minValue=0, maxValue=1000, integers=False)
```

Creation of synthetic urine metabolomic data:

```python
features = pd.read_csv("omic_features/urine_metabolites-2024-06-25.csv")['NAME']
data3 = simulate_data("metabolite", features, minValue=0, maxValue=1000, integers=False)
```

Creation of synthetic bulk RNASeq transcriptomic data:

```python
features = open("omic_features/hg38_ensembl_ids.txt").read().splitlines()
data4 = simulate_data("gene", features, minValue=0, maxValue=1000, integers=True)
```

Creation of synthetic gut microbiome data:

```python
features = open("omic_features/species.txt").read().splitlines()
data5 = simulate_data("species", features, minValue=0, maxValue=1000, integers=True)
```

Creation of synthetic germline short variants VCF data

The script for creating the synthetic VCF file can be found on Omilayers Github repository: `https://github.com/dkioroglou/omilayers/tree/main/synthetic_data/create_synthetic_vcf`

Each chromosome was created individually:

```
for i in {1..22} {X,Y,M};do python synthesize_vcf.py $i;done
```

And the synthetic VCF was created by joining all chromosomes:

```
for i in {1..22} {X,Y,M};do cat chr${i}.vcf >> simulated.vcf;done
```

Finally the resulted VCF was compessed creating the **simulated.vcf.gz** file.

S2 Retrieve stored layer

Retrieve stored layer for cohort features:

```
layer1 = omi.layers["cohort"].to_df()
```

Retrieve stored layer for blood metabolomics:

```
layer2 = omi.layers["blood_metas"].to_df()
```

Retrieve stored layer for urine metabolomics:

```
layer3 = omi.layers["urine_metas"].to_df()
```

Retrieve stored layer for RNASeq transcriptomics:

```
layer4 = omi.layers["rnaseq"].to_df()
```

Retrieve stored layer for gut microbiome:

```
layer5 = omi.layers["microbiome"].to_df()
```

Note: the method ”`.to_df()`” loads the stored layer as Pandas dataframe object.
Retrieve all rows for column ”SA100” from VCF:

```
sample = omi.layers["vcf"]["SA100"]
```

S3 Add new column to stored layer

Retrieve entire column from the RNASeq transcriptomics layer:

```
sampleRNASeq = omi.layers["rnaseq"]["SA100"]
```

Add new column to RNASeq transcriptomics layer:

```
omi.layers["rnaseq"]["SA101"] = sampleRNASeq
```

Retrieve entire column from the VCF layer:

```
sampleVCF = omi.layers["vcf"]["SA100"]
```

Add new column to VCF layer:

```
omi.layers["vcf"]["SA101"] = sampleRNASeq
```

S4 Perform row-based data retrieval

Row-based queries can be performed using the ”`.query()`” method that resembles the corresponding method from the Pandas API, or the ”`.select()`” method.
Retrieve row for columns ”ID”, ”SA010” and ”SA090” where chromosome is ”chr3” and position is ”100000”:

```
result = omi.layers['vcf'].query(f"CHROM == 'chr3' and POS == '100000'")[["ID", "SA010", "SA090"]]
```

Retrieve row for columns ”ID”, ”SA010” and ”SA090” where chromosome is ”chr22” and position is ”100000”:

```
result = omi.layers['vcf'].query(f"CHROM == 'chr22' and POS == '100000'")[["ID", "SA010", "SA090"]]
```

Retrieve rows for columns ”ID”, ”SA010” and ”SA090” where chromosome is ”chr15” and position is between ”50000” and ”50010”:

```
result = omi.layers['vcf'].query(f"CHROM == 'chr15' and POS BETWEEN 50000 AND 50010")[["ID", "SA010", "SA090"]]
```

Retrieve rows for columns ”ID”, ”SA010” and ”SA090” for all chromosomes where position is ”100000”:

```
result = omi.layers['vcf'].select(cols=["ID", "SA010", "SA090"], where='POS', values=100000)
```

S5 Perform row-based data retrieval in parallel

Retrieve rows for columns ”SA096”, ”SA098”, ”SA099” associated with 99 different positions of the chromosomes 1, 10 and 20:

```
import multiprocessing

def getSamples(chromo, pos):
    result = omi.layers['vcf'].query(f"CHROM == '{chromo}' and POS == {pos}")[['SA096', 'SA098', 'SA099']]
    return (chromo, pos, result.values)

queries = []
for i in [1,10,20]:
    for j in range(1, 100):
        queries.append((f"chr{i}", 100000+j))

with multiprocessing.Pool(processes=8) as pool:
    results = pool.starmap(getSamples, queries)
```

S6 Perform unittests

After installation:

```
pip install omilayers
```

The user can utilize the predefined unittests that are hosted on Omilayers Github repository to assess the reliability of Omilayers on his machine. To do so, the user needs to download the following files:

`https://github.com/dkioroglou/omilayers/blob/main/testing/tests_sqlite.py` for SQLite

`https://github.com/dkioroglou/omilayers/blob/main/testing/tests_duckdb.py` for DuckDB

To test the functionality of Omilayers for SQLite:

```
python -m unittests -v tests_sqlite.py
```

To test the functionality of Omilayers for DuckDB:

```
python -m unittests -v tests_duckdb.py
```
